# Excessive reassurance-seeking and anxiety among Chinese new urban older adults: the role of attention to negative information and resilience

**DOI:** 10.1186/s12877-025-06382-x

**Published:** 2025-09-02

**Authors:** Yang Sun, Cornelia Wrzus, Shaobo Lv

**Affiliations:** 1 Psychological Institute and Network Aging Research (NAR), Heidelberg University, Bergheimer Strasse 20, 69115 Heidelberg, Germany; 2 North China University of Science and Technology, Tangshan, China

**Keywords:** China, Rural-urban migration, Anxiety, Attention bias, Resilience

## Abstract

**Background:**

Policy-driven relocation of older adults from rural to urban areas in China is a significant stressor that often exacerbates anxiety. The mechanisms through which excessive reassurance seeking (ERS) heightens anxiety in this population remain poorly understood.

**Methods:**

A cross-sectional survey was conducted with 301 relocated older adults (aged 60–89 years; 52.5% women) in Jilin Province. Participants completed validated self-report measures assessing ERS, attention to negative information (ANI), resilience, and anxiety. Pearson correlations were used to assess bivariate relationships among the main variables. The PROCESS macro was employed to examine (a) the mediating role of ANI on the association between ERS and anxiety; (b) the moderating influence of resilience on the ERS-to-ANI path, thereby constituting a moderated-mediation model; and (c) the full-path robustness check of the model.

**Results:**

Participants reported clinically meaningful levels of anxiety, which were positively associated with excessive reassurance seeking. The association between ERS and anxiety was mediated primarily through ANI. Further analysis found that the indirect effect of ERS on anxiety through ANI was more pronounced in the presence of higher levels of resilience.

**Conclusion:**

ERS exacerbates anxiety mainly by amplifying negative attention bias. Even highly resilient individuals may experience more severe anxiety if they exhibit this attention bias. These findings emphasize that interventions should not only focus on fostering resilience, but also incorporate training to mitigate negative attention biases.

## Introduction

Over the past 40 years, China’s rapid economic growth and social change have been accompanied by an accelerating rate of urbanization. By the end of 2022, approximately 921 million Chinese residents lived in urban areas. During this period, approximately 12 to 16 million people migrated annually from rural to urban areas [1]. Scholars have described this large-scale internal migration as one of the most extensive in human history [2, 3]. At the same time, China’s growing ageing population is being reflected in the urbanization migration [4]. A distinctive feature of China’s urbanization process has been the government-driven land acquisition policies, which have relocated tens of millions of rural residents to urban areas [5]. These internal migrants are re-registered as urban residents and collectively resettled into government-provided compensatory urban communities [6]. Nearly half of these new urban residents are aged 60 or over, and are often referred to as “new urban older adults”, that is, emergent urban older residents who have been involuntarily relocated from rural areas to cities due to policy [7, 8].

Some studies indicate that involuntary relocation in later life may trigger or exacerbate relocation stress syndrome, suicidal ideation, anxiety, and depression [9–12]. These risks are particularly when older adults are placed in unfamiliar environments, where they must navigate new social networks and potential stigma. Anxiety is considered the most prevalent negative emotion in late adulthood, with studies showing that it is nearly twice as common as depression among older adults [13, 14]. Although multiple studies have explored potential mechanisms of anxiety among older adults [13, 15], research has yet to address why and how involuntary relocation may intensify anxiety in this population [16].

The loss of social networks due to relocation can have particularly negative effects on older adults [17], who are typically more motivated to maintain existing social relationships than younger individuals [18–20]. When confronted with abrupt disruptions in established social connections, older adults may experience threats to their self-integrity [21]. This can prompt excessive reassurance-seeking behaviours for social validation, potentially heightening interpersonal tensions and anxiety [22]. Based on this background, the present study primarily investigates the association between excessive reassurance-seeking and anxiety among Chinese new urban older adults, as well as the underlying psychological mechanisms.

### Excessive reassurance-seeking and anxiety

In recent years, excessive reassurance-seeking (ERS) has been widely discussed as a significant contributor on anxiety [23, 24]. ERS refer to a relatively stable tendency for individuals to continually seek reassurance from others that they are valuable, whether or not they have received this feedback repeatedly [25]. Initially, Coyne’s interpersonal theory [22] linked ERS to depression through mechanisms of social exclusion, and more recent studies have extended this framework to anxiety disorders [23]. Scholars propose two potential mechanisms underpinning the ERS-anxiety relationship. Specifically, ERS may strain interpersonal relationships, reducing or obstructing social support during critical or stressful periods [22, 26]. Cognitively, ERS might intensify anxiety by reinforcing individuals’ doubts about their self-efficacy [24]. Nevertheless, whether ERS contributes to anxiety among Chinese new urban older adults remains unclear. And, it is not yet known what additional factors may influence this relationship. Therefore, this study explores the association between ERS and anxiety in this population and hypothesises that ERS is positively associated with (*H1*).

### The potential mediating role of attention to negative information

In cognitive theory [27], attention to negative information (ANI) is an essential mechanism for sustaining and exacerbating negative emotions. Under stressful conditions such as involuntary relocation, individuals tend to adopt a bottom-up processing style, focusing disproportionately on negative information [28]. This attentional bias may serve as a bridging mechanism between ERS and anxiety in Chinese new urban older adults. For instance, older people who repeatedly seek reassurance without obtaining genuine satisfaction may consistently detect and amplify potential threats in their surroundings or interpersonal interactions, further exacerbating anxiety. Based on this, this study hypothesis that anxiety among Chines new urban older adults is influenced by attention to negative information during the reassurance-seeking process (***H2***). The mediating role of ANI is also examined.

### The potential moderating role of resilience

Individual differences in vulnerability to anxiety are often significant when coping with stressful situations (e.g., relocation). Resilience is widely regarded as a key protective psychological characteristic [29, 30]. Individuals with higher resilience typically maintain emotional balance or recover quickly when exposed to negative situations [31, 32]. To date, research findings on whether and how resilience moderates the ERS-ANI-anxiety pathway remain inconclusive. On the one hand, resilience may influence early cognitive processing stages; resilient individuals are typically better at regulating attention away from negative information [33]. This cognitive regulatory ability could buffer or weaken the initial ERS - ANI pathway, making individuals less prone to maladaptive cognitive patterns like excessive negative attention bias under stress. On the other hand, resilience might moderate later emotional processing stages, directly weakening the ANI-anxiety relationship by reducing emotional reactivity to negative cognitive biases [34]. Additionally, resilience could potentially moderate the direct ERS-anxiety relationship by mitigating the emotional distress associated with excessive reassurance-seeking behaviours [32, 35].

However, empirical evidence on these specific moderated pathways remains limited and inconsistent. Moreover, existing theoretical frameworks have not clearly identified which segments of the ERS-ANI-anxiety pathway resilience may moderate. Given this theoretical ambiguity and the exploratory nature of existing findings, the present study explicitly adopted an exploratory analytic strategy. Specifically, we examined the potential moderating role of resilience across multiple segments of the mediation model (ERS-ANI, ANI-anxiety, and ERS -anxiety) linking ERS to anxiety via ANI, without specific path was predetermined for moderation. Therefore, prior to data analysis, we proposed an exploratory hypothesis that resilience would moderate the indirect effect of ERS on anxiety through ANI among new urban older adults (***H3***).

### The present study

Although a growing body of research has examined the psychological effects of relocation, most studies have focused on international migrants or younger populations [36]. Psychological consequences among older adults undergoing involuntary, policy-driven relocation within China’s unique urbanization process have received limited scholarly attention. Guided by the interpersonal theory of excessive reassurance-seeking (ERS) and cognitive models of attentional bias [22, 27], we tested a moderated mediation model to examine the mechanisms underlying anxiety in this population. Specifically, we hypotheses that:

#### Hypothesis 1

*(****H1****)*: With higher ERS, new urban older adults experience more anxiety.

#### Hypothesis 2

*(****H2****)*: ANI mediates the association between ERS and anxiety in new urban older adults.

#### Hypothesis 3

*(****H3****)*: Higher resilience weakens the indirect effect of ERS on anxiety through ANI by moderating one or more of the paths among ERS-ANI, ANI-anxiety, and ERS -anxiety.

**Fig. 1:**
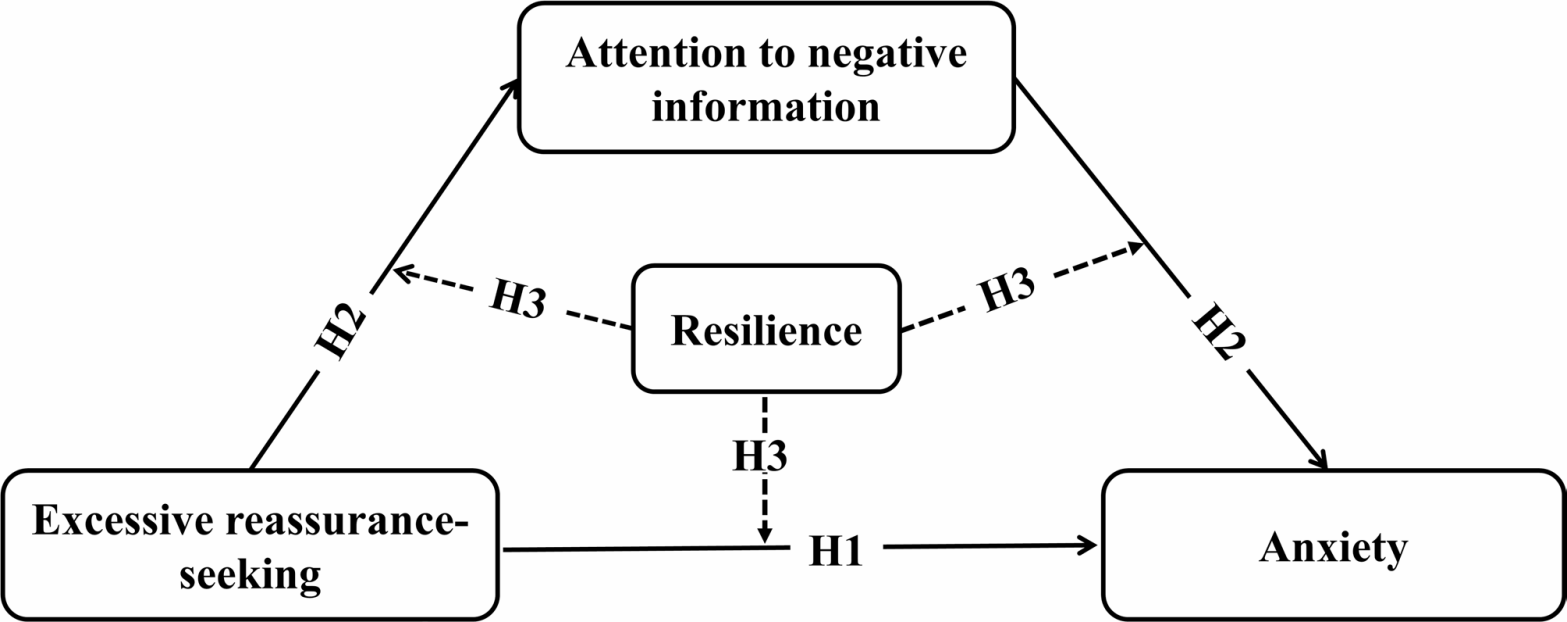
Hypothesised Moderated Mediation Model. Solid arrows represent the hypothesized direct and mediated effects. H1 posits a direct association between ERS and anxiety. H2 posits that ANI mediates the relationship between ERS and anxiety. Dashed arrows represent the exploratory moderation hypothesis (H3), suggesting that resilience may moderate one or more segments of the pathways linking ERS, ANI, and anxiety

The hypothesised conceptual model is illustrated in Fig. 1.

## Methods

### Participants

The target population comprised older adults who had been involuntarily relocated from rural to urban areas under government-driven urbanization policies and were residing in government-provided resettlement communities. Data collection conducted from May to August 2016 in a major city in southeastern Jilin Province, China.

To establish the sampling frame, we collaborated with two local committees responsible for managing these resettlement communities. These committees provided comprehensive, anonymized lists of all registered older adult residents who met our inclusion criteria. We then used a convenience sampling method on this defined sampling frame. All eligible individuals were approached and invited to participate until the target sample size was achieved. The inclusion criteria were as follows: (1) aged 60 or above, (2) relocated from rural to urban areas, (3) living in urban resettlement communities due to government-driven urbanization policies, and (4) free from severe cognitive impairment, with the capability to independently read and write simple text.

A total of 302 new urban older adults took part in the survey. One respondent was excluded for not meeting the age criterion, leaving a final analytic sample of 301 new urban older adults. To assess the adequacy of our study design, we performed a post-hoc power analysis in G*Power 3.1. Given the observed large effect size (*f*^*2*^ = 0.35), our sample size (*N* = 301) yielded a computed power of (1 - *β*) = 1.00, which exceeds common recommendations of 0.80 [37]. This results indicate that our sample size was sufficient to support subsequent analyses.

### Data collection procedures

This study was approved by the Ethics Committee of the North China University of Science and Technology (No. 17009), and conducted in accordance with the Declaration of Helsinki. Prior to the formal survey, a pilot study was conducted in February 2016 with three older adults from similar communities to assess wording clarity, skip-logic flow and font size. As this pilot served purely qualitative purposes, no statistical analysis was performed and the pilot data were not included in the main dataset.

The survey was conducted using self-administered questionnaires. Participants were informed about the purpose of the study and their rights, and provided written informed consent. Participants received sealed questionnaires and completed them either at home or in community-based senior activity centres. Due to participants’ advanced age and rural backgrounds, research assistants provided literacy support to 11 participants (3.64%), offering clarifications without influencing responses. At the end of the study, all participants received a non-monetary token of appreciation (e.g., a small potted plant).

After data collection, screening was conducted using the following exclusion criteria: questionnaires with more than 20% missing data (none excluded), migration duration exceeding 10 years (none excluded), and age under 60 (one case excluded).

### Measures

#### Anxiety (Dependent variable)

Anxiety was measured using the anxiety subscale of the simplified Chinese version of the Depression Anxiety Stress Scale (DASS-21) [38]. The subscale includes seven items assessing autonomic arousal, skeletal muscle effects, situational anxiety, and the subjective experience of anxious affect. The four-point scale ranges from 0 (does not apply) to 3 (applies totally). Scores are summed and then multiplied by two, resulting a total scores range from 0 to 42. According to the diagnostic criteria proposed by Lovibond, the scores range from 0 to 7 (normal); 8–9 (mild); 10–14 (moderate); 15–19 (severe); and 20 or more (extremely severe) [39]. In this study, the anxiety subscale exhibited excellent internal consistency (Cronbach’s α = 0.85), indicating strong measurement reliability.

#### Excessive reassurance-seeking (Predictor)

Excessive reassurance-seeking (ERS) was assessed using a validated 4-item subscale from the Depressive Interpersonal Relationships Inventory (DIRI-24, 20–23) [38, 39]. Items asked participants about their tendencies to seek comfort and their subjective feelings to feedback (e.g., “Do you frequently seek reassurance from the people you feel close to as to whether they really care about you?”). Responses were recorded on a 7-point Likert scale (0 = never and 6 = always). Participants’ ERS indexes were obtained by summing the scores of all items, with the total score ranging from 0 to 24. Higher total scores indicated a higher level of excessive reassurance-seeking. The ERS scale demonstrated acceptable internal consistency in this study (Cronbach’s α = 0.71).

#### Attention to negative information (Mediator)

Attention to negative information was measured using an 11-item scale derived from the Attention to Positive and Negative Information Scale (APNI) [40]. Participants rated how true each statement was from them (e.g., “I don’t forget when others do things that hurt me”) from 1 (very untrue of me) to 5 (very true of me). ANI scores were obtained by summing responses to all item, with higher scores indicating greater ANI. In this study, the ANI subscale showed acceptable internal consistency (Cronbach’s α = 0.61), which is falls within the acceptable range for short psychological scales [41].

#### Resilience (Moderator variable)

Resilience was measured using the Chinese version of the 10-item Connor-Davidson Resilience Scale (CD-RISC-10), a widely used instrument for assessing resilience in China [42]. The scale has been validated and meets established psychological standards for use in China [42, 43]. Items were answered on a 5-point scale ranging from 0 (not at all) to 4 (fully agree). The total score ranged from 0 to 40; higher scores indicated a higher resilience. The CR-RISC-10 showed excellent internal consistency in this study (Cronbach’s α = 0.88).

#### Demographic variables

Age (years), gender (male = 1, female = 2), and relocation duration (in years) were self-reported using single-item questions developed for this study.

##### Data analysis

All analyses were conducted using SPSS version 24.0. Prior to hypothesis testing, Harman’s single-factor test was conducted to assess possible common method bias. We also evaluated the normality of key variables to ensure the validity of our regression models. A Shapiro-Wilk test of residual indicated a significant deviation from normality (*p* <.05). This significant result of deviation might be partly attributable to the large statistical power for this test because, descriptive statistics showed skewness ranging from 0.03 to 0.33 and kurtosis ranging from − 0.78 to 0.53, both within acceptable thresholds [44]. Furthermore, our larger sample size (*N* = 301) allowed reliance on the central limit theorem, rendering subsequent parametric analyses acceptable [45].

After completing these preliminary checks, we performed descriptive analyses (*M*,* SD*) and Pearson correlation among key variables and relevant sociodemographic variables. To test our hypothesised mediation (Model 4) and moderated mediation (Model 7), we employed conditional process analysis using the PROCESS macro (version 4.2) developed by Hayes [46]. To verify the robustness of our moderated mediation results, we additionally performed analyses with Model 59, which simultaneously tested resilience as a moderator on all potential moderation paths.

Following Preacher et al. [47], the moderated mediation model was confirmed when the 95% confidence interval (CI) after 5,000 replicated simulations of the sample (*N* = 301) using the bootstrap method did not contain zero and when further simple slope analysis showed that the 95% CI of the mediated path with plus or minus one standard deviation of the moderating variable, one contained zero and one did not contain zero. Finally, we used the Johnson-Neyman technique based on the second-order variance method to plot conditional process curves with confidence intervals.

## Results

### Common method bias test

Since all data in the current study were collected through self-reported questionnaires, we conducted Harman’s single-factor test to assess the potential common method bias. The analysis revealed that nine factors had eigenvalues over one, and the first unrotated factor explained 19.06% of the total variance, significantly below the recommended threshold of 40% [48]. Thus, severe common method bias is unlikely to be a concern in the present study.

### Participant characteristics

Table 1 presents the sociodemographic and relocation characteristics of the 301 new urban older adults. The gender distribution was relatively balanced, with 52.5% of participants being female. Participants were mainly recent migrants, with an average of 3.3 years since relocation (*SD* = 1.78; range = 1–10). Most participants were in the younger-old age range, with a mean age of 65.9 years (*SD* = 5.02) and nearly four-fifths aged 60–69 years.

**Table 1:** Sociodemographic characteristics of participants (*N* = 301)

Characteristic	Statistic
Age (years)	
*M* (*SD*)	65.9 (5.02)
Age group	
60–69, *n* (%)	237 (78.7)
70–79, *n* (%)	59 (19.6)
≥ 80, *n* (%)	5.0 (1.7)
Gender	
Male, *n* (%)	143 (47.5)
Female, *n* (%)	158 (52.5)
Relocation Duration (years)	
*M* (*SD*)	3.3 (1.78)
Range	1–10

*M* = Mean; *SD* = Standard Deviation; *n* (%) = frequency and percentage

### Descriptive and correlation analysis

Descriptive statistics and bivariate zero-order correlations among all key study variables are presented in Table 2. The mean score for anxiety was 15.61, which meets the criteria for severe anxiety on the DASS-21 scale. No validated cut-off scores exist for the remaining variables (including resilience, ERS, and ANI); therefore, their means are reported for descriptive purposes only. In addition, the correlation matrices indicated that anxiety was higher with greater ERS, attention to negative information, and among the women. Anxiety was also higher with lower resilience and more recent the relocation, with higher ERS and ANI. Unexpectedly, participants with higher resilience also reported elevated levels of ERS and ANI. These results provided the basis for subsequent testing of the conditional process model.

**Table 2:** Descriptive statistics of study variables

Variable	M	SD	1	2	3	4
1. Anxiety	15.61	10.18	−0.13*			
2. Resilience	22.12	6.58	−0.02	−0.17**		
3. Excessive reassurance-seeking	8.32	4.56	0.07	0.13*	0.25**	
4. Attention to negative information	33.18	5.14	−0.09	0.21**	0.30**	0.30**

Significance levels: ^*^p <.05, ^**^p <.01

### Mediation effect analysis

The PROCESS macro (Model 4) was used to examine the mediation model (Hypothesis 1 and 2). The results showed that the indirect effect of ERS on anxiety via ANI was significant. The mediation effect index was 0.05 (95% CI [0.02, 0.10], *p* <.01). By contrast, the direct effect of ERS on anxiety was not significant (*β* = 0.08, *t* = 1.29, 95% CI [−0.04, 0.19]), suggesting that ANI plays a key role in the link between ERS and anxiety. The total effect of ERS on anxiety through ANI was significant (*β* = 0.13, *t* = 2.29, 95% CI [0.02, 0.24]). The indirect effect of ANI accounted for approximately 41.73% of the total effect, indicating that ERS contributes to increased anxiety primarily through its influence on ANI. These findings support Hypotheses 1 and 2.

### Moderated mediation model analysis

We tested for the potential moderating effect of resilience on the mediation pathway, beginning with the first stage (“ERS-ANI”). Using PROCESS macro’s Model 7, we found that this first stage was moderated by resilience (see Table 3). The results of the first regression equation showed that ERS had a significant effect on ANI and that resilience and the interaction of resilience and ERS both significantly predicted ANI (Table 3). Regression Eq. 2 indicated that ERS no longer had a significant direct effect on anxiety when ANI was included in the model, whereas ANI predicted anxiety (Table 3; right column).

**Table 3:** The relationship between excessive reassurance-seeking and anxiety: a moderated mediation model

Predictor Variable	Predicted Variable							
	ANI (mediator)				Anxiety (dependent variable)			
	β	SE	t	95% BCa CI	β	SE	t	95% BCa CI
ERS	0.14	0.06	2.50^*^	[0.03, 0.26]	0.08	0.06	1.29	[−0.04, 0.19]
ANI					0.18	0.06	3.12^**^	[0.07, 0.30]
Resilience	0.29	0.06	5.28^**^	[0.18, 0.40]				
ERS × Resilience	0.22	0.05	4.45^**^	[0.12, 0.32]				
*R* ^2^	0.19				0.05			
*F*(*df*)	23.83^**^ (3, 297)				7.56^**^ (2, 298)			

Significance levels: ^*^p <.05, ^**^p <.01

Further bias-corrected bootstrap analysis showed that the indirect effect of ERS on anxiety via ANI was moderated by resilience (Table 4). The mediating effect of ERS on anxiety through ANI was significant at average and high levels of resilience (+ 1 *SD*). However, it was non-significant for participants with low resilience (−1 *SD*). See Table 4 for detailed estimates at specific resilience levels.

Control analyses included gender, age, and relocation duration as covariates. Including these variables did not significantly alter the main results. In particular, no significant effects of age and gender were observed on the mediated ERS-ANI-anxiety pathway. Time since relocation showed a small association with anxiety (*β* = − 0.14, *SE* = 0.06, *p* =.02) but no significant effect on the first stage of the moderated mediator pathway. (See: https://osf.io/z8976/ for the control analyses).

Hayes [49] argued that if the moderating effect in the moderated mediation model exists only in one segment of the mediated path, the mediating effect being moderated should be a mathematically linear function of the moderating variable. The slope of this function is equal to the product of the regression coefficient of the interaction term of the independent and moderating variables on the mediating variable and the regression coefficient of the mediating variable on the dependent variable. Therefore, if the 95% CI of this slope does not contain zero, it can be shown that a moderated mediation effect exists and that the moderating variable only moderates one segment of the mediating path. Hayes referred to this slope as the index of moderated mediation, which can be tested by bootstrapping, a method also known as the product of coefficients method. It has now been confirmed by the results of many studies to have significant advantages over previous analysis methods and has gradually become more widely recognised by scholars in recent years [49]. The results of our analysis using the PROCESS macro (Table 4) showed that there was a significant index of moderated mediation (*β* = 0.04, *SE* = 0.02, 95% CI [0.01, 0.08]), which suggested that we did not need to carry out further model tests to determine whether other mediated paths also had a mediating effect.

To ensure maximum robustness and to provide comprehensive empirical evidence, we conducted an additional robustness check using PROCESS Model 59. This model simultaneously tests for moderation on all possible pathways. Consistent with the primary analysis, results indicated that only the first-stage moderation (ERS-ANI) was statistically significant, whereas moderation effects on the second-stage (ANI-Anxiety; *β* = 0.03, *p* =.58) and direct paths (ERS-Anxiety; *β* = 0.09, *p* =.09) remained non-significant. These findings empirically confirm that the moderated mediation effect operates exclusively through the ERS-ANI pathway. The full output for this robustness check is publicly available at: https://osf.io/z8976/.

**Table 4:** Estimated conditional indirect effect: ERS → ANI → anxiety at different values of resilience

Conditional indirect effect	β	Boot SE	Boot LLCI	Boot ULCI
−1 *SD* resilience	−0.01	0.02	−0.05	0.02
Mean resilience	0.03	0.01	0.01	0.06
+ 1 *SD* resilience	0.07	0.03	0.02	0.12
Index of moderated mediation	0.04	0.02	0.01	0.08

Conditional indirect effect = indirect (mediated) effect at the specified level of resilience

These results indicated that ANI mediated the relationship between ERS and anxiety, and this mediation was positively moderated by resilience at the first stage. The indirect effect (ERS-ANI-anxiety) was more pronounced with higher levels of resilience, which contradicted *H3*.

**Fig. 2:**
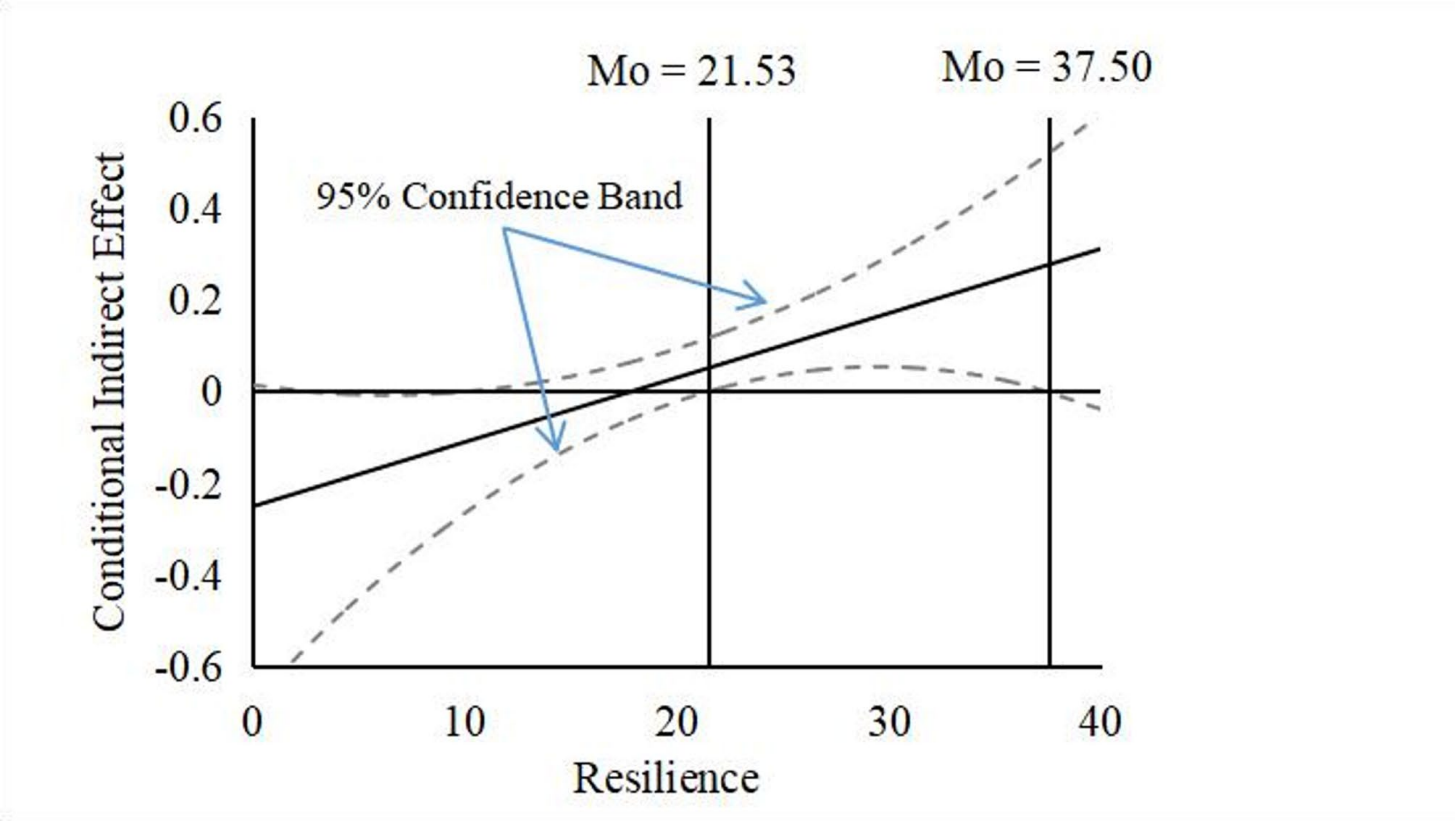
A Plot of the Indirect Effect of Excessive Reassurance Seeking on Anxiety via Attention to Negative Information in New Urban Older Adults Depending on Resilience (i.e., the Moderator). The horizontal line denotes an indirect effect of zero. The vertical line represents the boundary of the region of significance. The dashed lines indicate the 95% confidence bands

Figure 2 illustrates the conditional indirect effect, showing how resilience moderated the mediated role of ERS in the ERS-anxiety association. Following Preacher et al. [47], we used the Johnson-Neyman technique based on second-order variance to plot the conditional indirect effect with a 95% confidence interval band. According to Fig. 2, the indirect effect of ERS on anxiety via ANI was significant when the resilience of the participants was scored between 21.53 and 37.50 (out of 40).

## Discussion

The results of the current study support our research hypothesis (*H1* and *H2*). First, the results showed that some new urban older adults experience high levels of anxiety and ERS, and that greater ANI was associated with higher anxiety. This finding is consistent with prior studies among non-clinical younger adults, suggesting that ERS contributes to anxiety across different populations [23]. Rachman hypothesised that reassurance-seeking is the most common tendency and behaviour among individuals seeking security in negative situations [50]. Previous research has confirmed that relocation in later life can damage the physical and mental health of older people [10, 11, 16]. The involuntary relocation of new urban older adults due to policy mandates disrupted their existing, stable social support networks. This posed a great challenge given their declining physical and cognitive functioning. In the process, they often sought additional comfort from others in an effort to regain social support and psychological capital. This is consistent with previous research on the relationship between ERS and negative emotional disorders (e.g. depression). When individuals’ social support networks are disrupted and interpersonal relationships deteriorate, they are more likely to engage in ERS and experience feelings of hopelessness and depressed [51, 52].

Furthermore, our study has shown that ANI mediated the relationship between ERS and anxiety in new urban older adults. This finding supports previous research (involving U.S. samples mainly) on the relationship between ERS, ANI, and anxiety [28, 52–54]. Our findings suggest that ERS in new urban older adults does not necessarily cause anxiety; rather, it increases the potential for anxiety when there is an excessive focus on ANI.

**Fig. 3:**
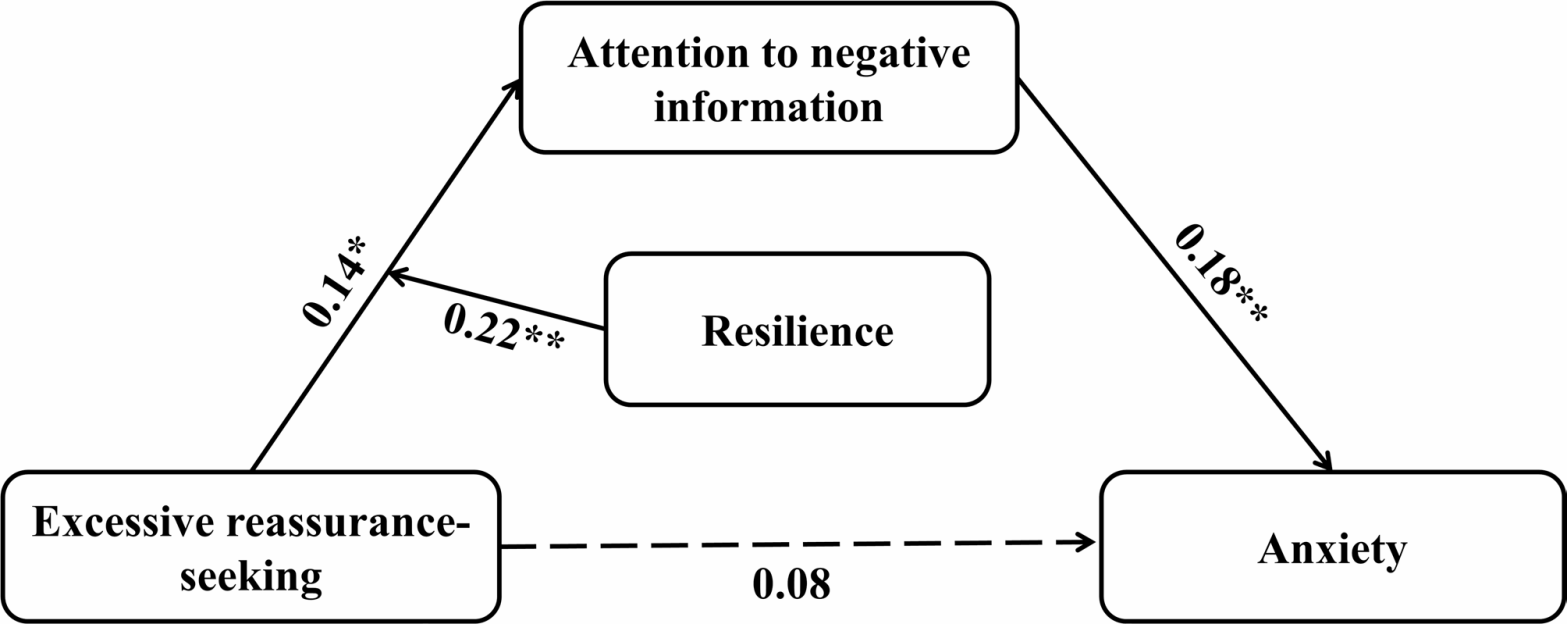
Summary of the Moderated Mediation Model of Anxiety Among New Urban Older Adults. Significance levels: *p <.05, **p <.01

Resilience is generally considered a psychological protective factor [35, 55]. In the present study, resilience partially moderated the mediating process of ERS-ANI-anxiety. Specifically, the moderating effect of resilience was observed only at the first stage of the model (see Fig. 3).

Contrary to H3, the results suggested that resilience increased the indirect effects of ERS on anxiety through ANI. While our findings differ from some previous studies [56, 57], resilience is a dynamic process with both protective and potentially risk-enhancing facets. Future researchers should explore not only the positive effects but also the developmental mechanisms and contexts in which resilience may backfire. One potential explanation for the stronger ERS-ANI-anxiety among high resilience individuals is that these older adults were more likely to rely on both internal and external resources to cope with psychological distress caused by later-life relocation. They may have found that their existing social support network was impaired as they entered an unfamiliar environment and faced possible stigmatisation. Such experiences might have led to stronger insecurity and increased attention to negative information during repeated reassurance-seeking, thereby creating a vicious cycle and further anxiety. This result is partly consistent with the Resilience Integration Model [58].

Additionally, our findings are consistent with the high-stress environment of involuntary relocation and its potential to activate interpersonal and cognitive vulnerabilities, as highlighted in the introduction section. From a theoretical perspective, the findings extend Coyne’s interpersonal theory by suggesting that ERS can have an impairing effect not only through social exclusion, but also through attention bias toward negative information [22]. And, the mediating role of ANI supports Beck et al.‘s cognitive theory, suggesting that selective attention to threat exacerbates negative emotions, in the current study anxiety [27]. Finally, this unexpected interaction with resilience suggests that the “protective” quality is not the only characteristic of resilience; under certain conditions, it may exacerbate maladaptive processes. This paradox corresponds with recent discussions on the complexity and dynamic of resilience [57, 59].

From a practical perspective, many involuntarily relocated older adults may be unaware that their ERS is a maladaptive coping strategy. Such behaviour may further damage their social networks and exacerbate anxiety, particularly as they tend to focus more on negative aspects of interpersonal interactions. To mitigate anxiety, older adults can be supported in strengthening and re-establishing meaningful social connections, while also learning strategies to reduce selective attention to negative information. Therefore, policymakers and mental health practitioners should cooperate in designing community programmes that foster adaptive coping skills and provide sustainable social support. Interestingly, the paradoxical role of resilience indicates that some older adults who appear resourceful may still struggle to cope effectively, highlighting the importance of personalised assessments and interventions.

The present study has both strengths and weaknesses. First, it examined the underexplored psychological effects of involuntary relocation among older adults, using well-validated measures and a gender-balanced sample. The results may also inform policymakers and support older adults in other countries who are involuntarily retirement homes. Second, due to the cross-sectional design of the study, it is not possible to draw any causal inferences about the relationship between ERS and anxiety in new urban older adults. It is equally plausible that higher levels of anxiety lead to ERS and that bidirectional effects exist. Follow-up investigations and additional experimental designs will be needed if the strengths of the specific directional effects of the conditional process model proposed in the present study are to be disentangled. Although reliance on self-report questionnaires is common in ageing research, it carries the risk of method bias. Mixed methods (e.g., involving the use of other reports of ERS and indirect measures of ANI) may help to mitigate this concern. Finally, the participants were drawn from a convenience sample in the northeastern region of China, so our results may not be generalizable to all relocated older adults and to other regions. Self-selection bias is also possible: either (a) individuals experiencing particularly high burdens, or (b) those with fewer adjustment problems, may have declined to participate. However, the range and standard deviations of our main variables (e.g., anxiety, resilience) indicate that a relatively broad range of participants was included. Still, regional variation in economic and resettlement policies may lead to differences in new urban older adults’ adaptability and their levels of ERS, resilience, and anxiety. Future studies should consider recruiting participants from diverse geographical regions to enhance the generalizability of their findings.

## Conclusion

This study found that Chinese new urban older adults who experienced later-life relocation tended to exhibit higher anxiety, with ERS and ANI identified as significant contributing factors. By reducing ANI, it may be possible to reduce the risk of anxiety arising from ERS. In addition, the findings indicate that high levels of resilience might not be beneficial in all circumstances. Indeed, resilience can sometimes be associated with detrimental outcomes, such as increased attention to negative information. The findings also suggest that older adults with high levels of resilience may pay more attention to negative information during reassurance-seeking, thereby increasing/causing their anxiety. Therefore, psychological interventions should also consider targeting older adults with high resilience, as they may be at unexpected risk of anxiety due to maladaptive attentional patterns.

## Data Availability

All data included in this study can be obtained by contacting the corresponding author. Requests for data will be processed by the corresponding author, subject to an assessment of the legitimacy of the request, and compliance with any applicable ethical and legal requirements.

## Abbreviations

ERS: Excessive reassurance-seeking
ANI: Attention to negative information
